# An investigation of factors predicting the type of bladder antimuscarinics initiated in Medicare nursing homes residents

**DOI:** 10.1186/s12877-017-0690-2

**Published:** 2017-12-28

**Authors:** Daniela C. Moga, Qishan Wu, Pratik Doshi, Amie J. Goodin

**Affiliations:** 1 Department of Pharmacy Practice and Science, College of Pharmacy; Department of Epidemiology, College of Public Health; Sanders-Brown Center on Aging; Institute for Pharmaceutical Outcomes and Policy, University of Kentucky, 789 S Limestone Street, Room 241, Lexington, KY 40536 USA; 2 Department of Pharmacy Practice and Science, Institute for Pharmaceutical Outcomes and Policy, College of Pharmacy, University of Kentucky, Lexington, KY USA; 3 Department of Pharmaceutical Outcomes and Policy, College of Pharmacy, University of Florida, Gainesville, FL USA

**Keywords:** Bladder antimuscarinics, Nursing homes, Elderly incontinence

## Abstract

**Background:**

To examine factors predicting type of bladder antimuscarinics (BAM) initiated in nursing home (NH) residents.

**Methods:**

Incident BAM initiators following NH admission were identified by constructing a retrospective cohort from Medicare files and Minimum Data Set (MDS). Participants included all residents 65 years and older admitted in Medicare-certified NH between January 1, 2007 and December 31, 2008 who were prescribed BAM and had continuous Medicare (Part A, B, and D) enrollment. Patient characteristics, medications, and comorbidities were derived from Medicare enrollment and claims. NH characteristics and health status were derived from MDS assessments. The outcome was defined as type of BAM initiated after admission (selective, non-selective extended release, non-selective immediate release). Multinomial logistic regression using generalized estimating equation methodology determined which factors predicted the type of BAM initiated.

**Results:**

Twelve thousand eight hundred ninety-nine NH residents initiating BAM therapy were identified; 13.38% of new users were prescribed selective BAM, 45.56% non-selective extended release, and 41.07% non-selective immediate release medications. In both sexes, significant predictors of BAM included region of nursing home, body mass index, cognitive performance score, frailty measures, activities of daily living, and measures of bladder continence. In women, history of fracture and fall-related injuries were significant predictors of type of BAM use, while race and indicators of balance were significant predictors of type of BAM use in men. Non-pharmacological continence management strategies were not predictive of type of BAM initiation.

**Conclusions:**

Several factors are important in predicting type of BAM initiation in both women and men, but other factors are sex-specific. Some observed factors predicting the type of BAM initiated, such as other medications use, body mass index, or provider-related factors are potentially modifiable and could be used in targeted interventions to help optimize BAM use in this population.

**Trial registration:**

Not applicable.

**Electronic supplementary material:**

The online version of this article (doi:10.1186/s12877-017-0690-2) contains supplementary material, which is available to authorized users.

## Background

Urinary incontinence is defined as the involuntary loss of urine [1], or the loss of urinary bladder control, and includes stress, urgency and mixed incontinence [2]. Urgency incontinence, which is the incontinence associated with a strong desire to void, comprises the most common underlying type of urinary incontinence in older persons. Prevalence estimates of urinary incontinence range from 43 to 77%, making urinary incontinence one of the most common conditions to affect nursing home residents [3]. There are several negative consequences for those living with this condition. Urinary incontinence is associated with an increased risk of falls, fractures, or bacterial infections [4]. Previous studies have shown that urinary incontinence increased the number of hospitalizations by 30 to 50% [5], and negatively impacted quality of life (QOL) in nursing home residents [6]. Additionally, the economic costs associated with managing urinary incontinence are significant [7, 8].

To date, available treatment options for urinary incontinence result in various degrees of symptom management, rather than a curative effect. Selecting the appropriate urinary incontinence treatment strategy depends on the type of incontinence, its severity and the underlying cause, and often time, different non-pharmacological and/or pharmacological options are used in combination to provide the best symptom management for a particular patient. The available non-pharmacological alternatives include behavioral therapies (i.e., bladder training, double voiding, fluid consumption, healthy lifestyle changes, or scheduled toilet trips, pelvic floor muscle exercises), electrical stimulation, absorbent pads and catheters, or surgical interventions [9–14]. Pharmacological interventions are available for urgency and mixed incontinence, with bladder antimuscarinic (BAM) drugs at the forefront, often time in addition to behavioral strategies. These drugs have an antagonistic effect on the muscarinic receptors in the bladder detrusor muscle that provide beneficial effects on urgency or mixed urinary incontinence management [15, 16]. Clinical trials have shown the effectiveness of these drugs in reducing incontinence episodes [17–23]; however, it should be noted that some of these trials were conducted in younger populations or outside of the long-term care environment, and findings may not be generalizable to older patients living in nursing homes [18–20]. In addition to the bladder muscarinic receptors, the five different muscarinic receptors (M1–M5) [24] are widespread throughout the body resulting in various undesirable effects after BAM drugs, especially when non-selective agents are initiated. Some of these effects are bothersome and may be associated with treatment discontinuation [25, 26]. Other adverse effects, such as falls, fractures, or cognitive impairment, have been associated with significant risks, including an increased mortality [27–29]. To our understanding, there is limited information available on factors influencing BAM therapy selection in the nursing home population and previous investigations raised question on whether drug therapy for urinary incontinence is optimally used in long-term care [30]. It is important to understand these factors given the non-curative effects and adverse event potential of BAM, especially those factors that are modifiable and could be targeted in future interventions aimed to optimize medication use. Furthermore, gathering information on factors associated with BAM initiation would be important for future comparative effectiveness studies by informing the selection of appropriate strategies for confounding control when using observational data. Additionally, there is evidence to suggest that nonselective BAM agents may be associated with stronger negative effects on cognitive function than selective BAM agents [31–33], but controversy still exists whether all or only some BAM pose these potential risks [34]. As many of the studies investigating the link between BAM use and adverse events were conducted with small samples, in community dwelling and/or younger populations, these future studies are needed to address the existing controversies regarding not only differences in risks with different BAM, but also to establish the clinical evidence for the nursing homes population. Therefore, the objective of this study was to identify factors predicting the type of BAM initiated in long-term care residents in Medicare nursing homes throughout the US. In addition, considering the important sex-differences related to urinary incontinence prevalence, symptomatology, and treatment seeking behavior, we also investigated whether there are differences in predictors between women and men.

## Methods

### Data sources and study population

**Fig. 1:**
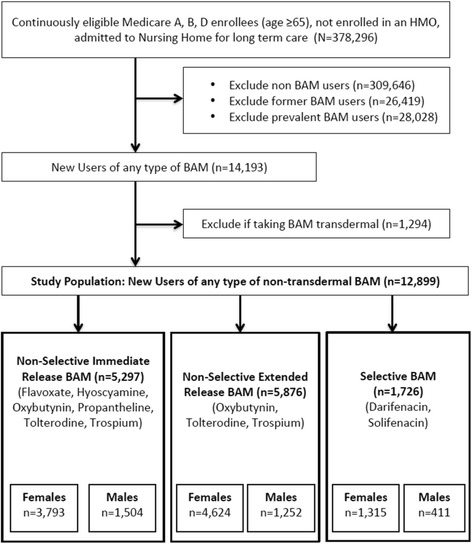
Study Sample Selection: Flowchart of Inclusion/Exclusion Criteria

Study design and all of the analyses were conducted based on an a priori specified protocol approved by the Institutional Review Board at the University of Kentucky. Due to the retrospective nature of the design, the Institutional Review Board granted the investigators a waiver of informed consent. For this study we linked Medicare claims data with Minimum Data Set (MDS) assessments. The linked dataset contained enrollment information, inpatient and pharmacy claims, and MDS assessment data collected in between January 1, 2007 through December 31, 2008. We included patients 65 years or older who were continuously eligible for Medicare Part A, B, and D, but no enrollment in a Health Maintenance Organization (HMO) plan and were admitted to any of the Medicaid and/or Medicare certified long-term care facilities, all of which conduct MDS assessments. We also required an admission followed by a minimum of one quarterly MDS assessment to ensure that only long-term stays were included in the analyses [35]. If a patient had more than one long term stay in a nursing home during our study period, then only the first stay was examined in this study. Figure 1 provides a detailed outline of the exclusion and inclusion criteria for the study sample.

**Fig. 2:**
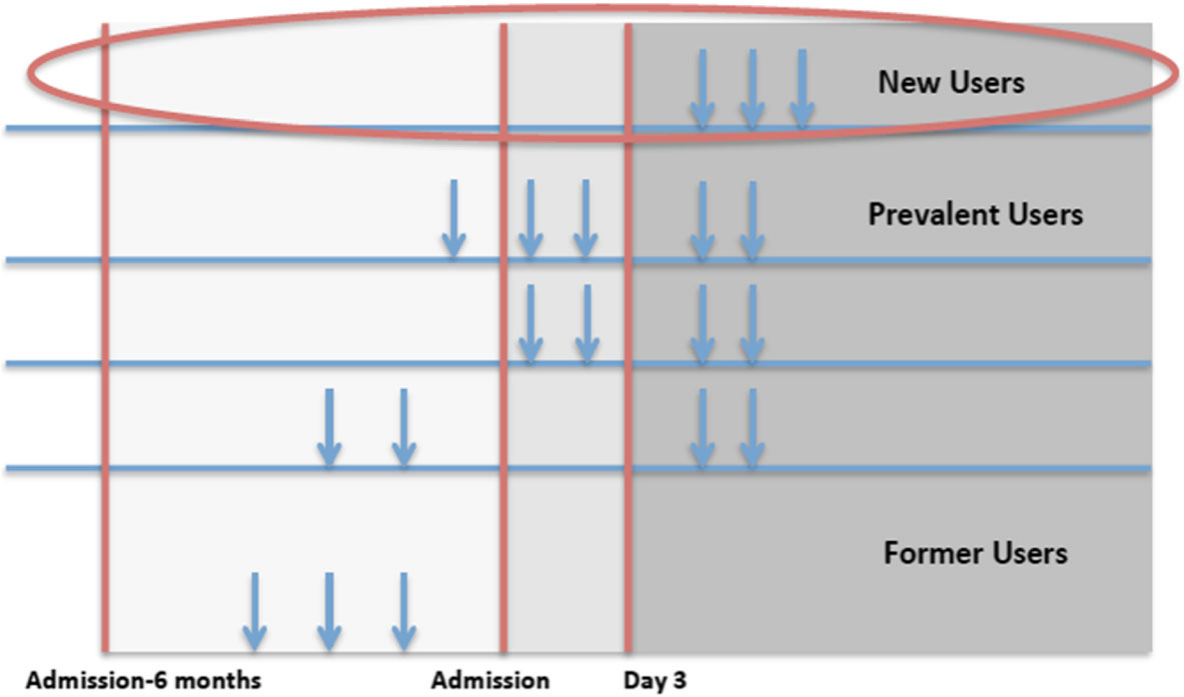
Defining New Bladder Antimuscarinics Users

Patients who did not receive prescriptions for BAM at any point in the study period were defined as “non BAM users” and were excluded from the analysis. Those with at least one pharmacy claim for any BAM (i.e., flavoxate, hyoscyamine, oxybutynin, propantheline, tolterodine, trospium, darifenacin, solifenacin) were considered BAM users and were further categorized as incident, prevalent, or former users (see Fig. 2). Non-selective immediate release BAM formulations included: flavoxate, hyocyamine, oxybutnynin, propantheline, tolterodine, and trospium. Non-selective extended release BAM formulations included: oxybutynin, tolterodine, and trospium. Selective BAM formulations included darifenacin and solifenacin. Our analyses included only those identified as incident (or new) users of a non-transdermal formulation who did not have BAM therapy prior to nursing home admission. Prevalent users were defined as (1) those with a minimum of one pharmacy claim for a BAM during their nursing home stay that also had at least one claim before nursing home admission, or (2) those with a pharmacy claim for BAM during their nursing home stay, but with insufficient medical history in the dataset (i.e., 6 months), or (3) those with a pharmacy claim for BAM during the first 3 days of their nursing home stay, regardless of the history in the dataset [36]. Patients who had received a prescription for BAM at any point in the claims data look-back period (from January 1, 2007 until nursing home admission), but not during nursing home stay were considered former users. Patients who were prescribed BAM at least 3 days or more following nursing home admission in the study period, and also had a minimum of 6 months of medical history in our dataset were defined as the “new user” [36]. The date of the first pharmacy prescription claim for a BAM was considered the index date for that person. New BAM users were further sub-divided into three categories based on muscarinic receptors’ selectivity and formulation; specifically, we grouped them as selective BAM medications, and either extended release (ER) non-selective BAM or immediate release (IR) non-selective BAM. Additional file 1: Table S1 includes a list of all BAM medications analyzed in this study, along with a summary of their muscarinic effects, other important drug characteristics and their clinical relevance [34, 37–45]. The last MDS assessment record prior to the index date along with Medicare enrollment files and medical and pharmacy claims in the 6 months period preceding this date were used to determine baseline characteristics used in the analysis.

### Variables

The outcome of interest for the study was defined as the type of BAM at initiation. For our analysis, the reference category consisted of patients who were new users of non-selective IR BAM. Potential predictors of type of BAM initiation were identified from enrollment files, claims, and from MDS assessments. The list of potential predictors included (1) socio-demographic characteristics (age at BAM initiation, sex, race, nursing home geographic region within one of the four major Census tracts, body mass index [BMI]); (2) cognitive measures (patient Cognitive Performance Score at BAM initiation) [46, 47]; (3) functional measures (Changes in Health, End-Stage Disease and Symptoms and Signs [CHESS] score [48], Activities of Daily Living [ADL] [49], balance, and gait performance); (4) bladder and bowel incontinence measures; (5) measures of non-pharmacological treatment for urinary incontinence (any scheduled toileting plan, indwelling catheter, or pads/briefs used); (6) comorbidities either identified based on the Elixhauser algorithm [50] or from the last MDS assessment prior to BAM initiation (hemiplegia/hemiparesis, quadriplegia, aphasia, cerebral palsy, multiple sclerosis, Parkinson’s disease, bipolar disorder, and schizophrenia); (7) anticholinergic load measured using the anticholinergic drug scale, with removal of BAM [51]; and (8) concomitant medications, including: cognitive enhancers, antiparkinson medications, diuretics, antipsychotics, antivertigo medications, beta blockers, anxiolytic sedatives, ACE inhibitors, antidepressants, ARBs, alpha blockers, anticonvulsants, vasodilators, and benzodiazepines. Table 1 contains a list of study variables with their respective data sources.

**Table 1:** Identification and Definition of Study Variables

Variable Group	Data Source	Identification and/or Definition
Socio-demographic Characteristics	Enrollment Files, Claims, Minimum Data Set	Patient age at BAM initiation, gender, race, nursing home geographic region, calculated Body Mass Index (BMI)
Cognitive Performance Score [46, 47]	Minimum Data Set	Categories were collapsed as follows: Intact, Borderline Intact/Mild Impairment, Moderate Impairment, Moderate-severe/Severe/Very Severe Impairment.
Behavioral Score [56]	Minimum Data Set	Total score includes sum of score of five items, where every item is measured on a scale from 0 (behavior not present in past 7 days) to 3 (behavior is daily), with a total score possibility between 0 and 15 points. The items include wandering (item E4a), verbally abusive behavior (item E4b), physically abusive behavior (item E4c), socially inappropriate or disruptive behavioral symptoms (item E4d), and resistance to care (item E4e).
Changes in Health, End-Stage Disease and Symptoms and Signs (CHESS) [48]	Minimum Data Set	Categories were collapsed as follows: Score of 0, Score of 1 or 2, Score of 3, Score of 4 or 5.
Activities of Daily Living [49]	Minimum Data Set	Total score range from 0 to 28.
Elixhauser Comorbidities [50]	Minimum Data Set	Diagnosis indicators for: congestive heart failure, cardiac arrhythmia, peripheral vascular disorders, hypertension (uncomplicated and complicated), diabetes (uncomplicated and complicated), renal failure, AIDS/HIV, weight loss, paralysis, other neurological disorders, psychoses, depression, blood loss anemia.
Other Comorbidities	Minimum Data Set, Claims	Diagnosis of: hemiplegia/hemiparesis, paraplegia, quadriplegia, aphasia, cerebral palsy, multiple sclerosis, Parkinson’s disease, manic depression/bipolar disease, schizophrenia.
Continence-Related Measures	Minimum Data Set	Bladder continence (item H1B), bowel continence (item H1A), bowel elimination patterns (items H2A, H2B, H2C, H2D), continence management (scheduled toileting plan [item H3A], bladder retraining program [item H3B], external catheter [item H3C], indwelling catheter [item H3D], intermittent catheter [item H3E], pads/briefs used [item H3G], no appliance or program [item H3J]).
Infection	Minimum Data Set	Diagnosis of urinary tract infection in last 30 days (item I2J).
Balance and Gait	Minimum Data Set	Balance while standing (item G3A), balance while sitting (item G3B), unsteady gait (item J1 N), fell in past 30 days (item J4A), fell in past 31–180 days (item J4B), hip fracture in last 180 days (item J4C), other fracture in last 180 days (item J4D).
Total Medications and Concomitant Medications	Claims	Total number of medications were counted for last 7 days and categorized as: 0 medications, 1 to 5, 6 to 10, or >10 medications. Concomitant medication classes included: calcium channel blockers, cognitive enhancers, antiparkinson, diuretics, antipsychotics, antivertigo, beta blockers, anxiolytic sedative/hypnotics, ACE inhibitors, antidepressants, ARB, alpha blockers, anticonvulsant, vasodilators, benzodiazepines.
Anticholinergic Burden/ADS Score [51]	Claims	Calculated from concomitant medications after removal of prescribed BAM medications.

### Statistical analysis

Frequencies and descriptive statistics of patient characteristics were calculated for the whole study sample of new BAM users and for new users by type of BAM. Multinomial logistic regression via generalized estimating equations (GEE) was employed to determine the factors predicting type of BAM initiation. Predictors were selected for inclusion in the multivariable analysis using a manual backward elimination procedure. After including sex as potential predictor, we also conducted the analyses using stratification by sex to determine whether there were sex-specific differences in factors predicting type of BAM at initiation.

Approximately 4.39% of the total study population’s records contained at least one missing value, primarily among CHESS scores and BMI. To address the potential for bias, we conducted sensitivity analyses to determine whether there were any differences in the type of BAM uptake between those with complete information and those with missing data. Specifically, we used imputation techniques to assign that variable’s mode to the missing value (e.g., if a patient record had a missing CHESS score, the mode of the CHESS variable was imputed for that patient record). Results of the model using the imputed data were compared to the original results to test robustness of the original model.

Goodness of fit was assessed for each model via Pearson tests for overall fit and Likelihood Ratio tests for joint significance of all predictors. Validation for each model was conducted by creating a confusion matrix (aka cross classification table) using observed and predicted values calculated from the fitted models. The percent match, meaning proportion of correctly classified variables, was then reported for each model. All analysis was conducted in SAS v9.4 [52].

## Results

Treatment with BAM was initiated by 12,899 nursing home residents; of these, 1726 (13.38%) were prescribed selective BAM, 5876 (45.56%) were prescribed non-selective ER BAM, and 5297 (41.07%) were prescribed non-selective IR BAM. New BAM users groups were similar in demographic characteristics such as age and race (Table 2); however, fewer non-selective IR BAM users were bowel continent when compared to non-selective ER BAM users and selective BAM users. Also, non-selective IR BAM users demonstrated less ability to maintain balance while sitting when compared with other BAM users. Lastly, there were geographic prescribing differences among the types of BAM, with fewer non-selective IR BAM among residents users in nursing homes located in the Midwest region and a greater proportion of IR BAM users in the northeast region.

**Table 2:** Study Sample Characteristics at Baseline

	New Non-Selective IR Users (*n* = 5297)	New Non-Selective ER Users (*n* = 5876)	New Selective Users (*n* = 1726)	Total New Users (*n* = 12,899)
Age				
65–74	942 (17.8%)	1046 (17.8%)	309 (17.9%)	2297 (17.8%)
75–84	1959 (37.0%)	2318 (39.4%)	651 (37.7%)	4928 (38.2%)
85 and Older	2396 (45.2%)	2512 (42.8%)	766 (44.4%)	5674 (44.0%)
Sex				
Female	3793 (71.6%)	4624 (78.7%)	1315 (76.2%)	9732 (75.4%)
Male	1504 (28.4%)	1252 (21.3%)	411 (23.8%)	3167 (24.6%)
Race				
White	4669 (88.1%)	5278 (89.8%)	1561 (90.4%)	11,508 (89.2%)
Black	459 (8.7%)	427 (7.3%)	115 (6.7%)	1001 (7.8%)
Other	169 (3.2%)	171 (2.9%)	50 (2.9%)	390 (3.0%)
Nursing Home Region				
Midwest	1744 (32.9%)	2695 (45.9%)	744 (43.1%)	5183 (40.2%)
Northeast	1500 (28.3%)	1168 (19.9%)	285 (16.5%)	2953 (22.9%)
South	1705 (32.2%)	1615 (27.5%)	613 (35.5%)	3933 (30.5%)
West	348 (6.6%)	398 (6.8%)	84 (4.9%)	830 (6.4%)
Body Mass Index				
Underweight	573 (10.9%)	422 (7.2%)	100 (5.8%)	1095 (8.5%)
Normal	2304 (43.9%)	2322 (39.7%)	674 (39.3%)	5300 (41.4%)
Overweight	1357 (25.8%)	1614 (27.6%)	515 (30.0%)	3486 (27.2%)
Obese	1018 (19.4%)	1487 (25.4%)	428 (24.9%)	2933 (22.9%)
Cognitive Performance Score				
Intact	978 (18.5%)	1355 (23.1%)	384 (22.3%)	2717 (21.1%)
Borderline intact/Mild impairment	1661 (31.4%)	2286 (39.0%)	698 (40.5%)	4645 (36.1%)
Moderate impairment	1598 (30.2%)	1826 (31.1%)	523 (30.3%)	3947 (30.7%)
Moderate-severe/Severe/Very Severe impairment	1046 (19.8%)	402 (6.8%)	119 (6.9%)	1567 (12.2%)
Mean Behavioral Score (SD)	0.6 (1.42)	0.4 (1.15)	0.4 (1.12)	0.5 (1.27)
CHESS				
Score of 0	1397 (27.5%)	1761 (30.9%)	560 (33.6%)	3718 (29.9%)
Score of 1 or 2	2984 (58.7%)	3418 (60.0%)	943 (56.6%)	7345 (59.0%)
Score of 3	552 (10.9%)	432 (7.6%)	133 (8.0%)	1117 (9.0%)
Score of 4 or 5	152 (3.0%)	85 (1.5%)	30 (1.8%)	267 (2.1%)
Mean Activities of Daily Living (SD)	17 (7.13)	14.5 (6.59)	14.0 (16.7)	15.5 (6.95)
Elixhauser Comorbidities				
Congestive Heart Failure	1291 (24.4%)	1204 (20.5%)	352 (20.4%)	2847 (22.1%)
Cardiac Arrhythmia	1233 (23.3%)	1223 (20.8%)	345 (20.0%)	2801 (21.7%)
Peripheral Vascular Disorders	388 (7.3%)	434 (7.4%)	114 (6.6%)	936 (7.3%)
Hypertension, Uncomplicated	2227 (42.0%)	2723 (46.3%)	744 (43.1%)	5694 (44.1%)
Hypertension, Complicated	563 (10.6%)	519 (8.8%)	164 (9.5%)	1246 (9.7%)
Diabetes, Uncomplicated	1130 (21.3%)	1215 (20.7%)	346 (20.0%)	2691 (20.9%)
Diabetes, Complicated	290 (5.5%)	323 (5.5%)	78 (4.5%)	691 (5.4%)
Renal Failure	669 (12.6%)	572 (9.7%)	192 (11.1%)	1433 (11.1%)
AIDS/HIV	1 (0.0%)	1 (0.0%)	1 (0.1%)	3 (0.0%)
Weight Loss	504 (9.5%)	311 (5.3%)	87 (5.0%)	902 (7.0%)
Paralysis	225 (4.2%)	220 (3.7%)	49 (2.8%)	494 (3.8%)
Other Neurological Disorders	733 (13.8%)	648 (11.0%)	189 (11.0%)	1570 (12.2%)
Psychoses	281 (5.3%)	314 (5.3%)	86 (5.0%)	681 (5.3%)
Depression	835 (15.8%)	968 (16.5%)	300 (17.4%)	2103 (16.3%)
Blood Loss Anemia	114 (2.2%)	123 (2.1%)	29 (1.7%)	266 (2.1%)
Other Comorbidities				
Hemiplegia/hemiparesis	451 (8.5%)	420 (7.1%)	111 (6.4%)	982 (7.6%)
Paraplegia	24 (0.5%)	25 (0.4%)	8 (0.5%)	57 (0.4%)
Quadriplegia	21 (0.4%)	8 (0.1%)	1 (0.1%)	30 (0.2%)
Aphasia	243 (4.6%)	96 (1.6%)	26 (1.5%)	365 (2.8%)
Cerebral Palsy	11 (0.2%)	7 (0.1%)	1 (0.1%)	19 (0.1%)
Multiple Sclerosis	31 (0.6%)	32 (0.5%)	12 (0.7%)	75 (0.6%)
Parkinsons Disease	399 (7.5%)	389 (6.6%)	143 (8.3%)	931 (7.2%)
Manic Depression/Bipolar Disease	128 (2.4%)	162 (2.8%)	39 (2.3%)	329 (2.6%)
Schizophrenia	121 (2.3%)	129 (2.2%)	40 (2.3%)	290 (2.2%)
Bladder Continence				
Continent	2172 (41.0%)	2431 (41.4%)	706 (40.9%)	5309 (41.2%)
Usually Continent	515 (9.7%)	748 (12.7%)	211 (12.2%)	1474 (11.4%)
Occasionally Incontinent	521 (9.8%)	873 (14.9%)	248 (14.4%)	1642 (12.7%)
Frequently Incontinent	918 (17.3%)	1221 (20.8%)	364 (21.1%)	2503 (19.4%)
Incontinent	1170 (22.1%)	603 (10.3%)	197 (11.4%)	1970 (15.3%)
Bladder Continence Management				
Scheduled Toileting Plan	1369 (25.8%)	1616 (27.5%)	448 (26.0%)	3433 (26.6%)
Bladder Retaining Program	58 (1.1%)	70 (1.2%)	12 (0.7%)	140 (1.1%)
External Catheter	11 (0.2%)	11 (0.2%)	1 (0.1%)	23 (0.2%)
Indwelling Catheter	1133 (21.4%)	855 (14.6%)	221 (12.8%)	2209 (17.1%)
Intermittent Catheter	57 (1.1%)	58 (1.0%)	20 (1.2%)	135 (1.0%)
Pads/Briefs Used	3376 (63.7%)	3306 (56.3%)	1002 (58.1%)	7684 (59.6%)
No Appliance or Program	1172 (22.1%)	1755 (29.9%)	496 (28.7%)	3423 (26.5%)
Bowel Continence				
Continent	2544 (48.1%)	3821 (65.0%)	1113 (64.5%)	7478 (58.0%)
Usually Continent	461 (8.7%)	520 (8.9%)	185 (10.7%)	1166 (9.0%)
Occasionally Incontinent	307 (5.8%)	393 (6.7%)	100 (5.8%)	800 (6.2%)
Frequently Incontinent	565 (10.7%)	494 (8.4%)	134 (7.8%)	1193 (9.3%)
Incontinent	1417 (26.8%)	647 (11.0%)	194 (11.2%)	2258 (17.5%)
Bowel Elimination Patterns				
Regular	4154 (78.4%)	45,98 (78.3%)	1342 (77.8%)	10,094 (78.3%)
Constipation	494 (9.3%)	557 (9.5%)	166 (9.6%)	1217 (9.4%)
Diarrhea	277 (5.2%)	228 (3.9%)	70 (4.1%)	575 (4.5%)
Fecal Impaction	4 (0.1%)	3 (0.1%)	2 (0.1%)	9 (0.1%)
Urinary Tract Infection in Last 30 Days	1210 (22.8%)	1285 (21.9%)	384 (22.2%)	2879 (22.3%)
Vision				
Adequate	3588 (68.2%)	4196 (71.5%)	1240 (71.8%)	9024 (70.2%)
Impaired	935 (17.8%)	1074 (18.3%)	314 (18.2%)	2323 (18.1%)
Moderately Impaired	330 (6.3%)	347 (5.9%)	97 (5.6%)	774 (6.0%)
Highly Impaired	318 (6.0%)	172 (2.9%)	51 (3.0%)	541 (4.2%)
Severely Impaired	93 (1.8%)	81 (1.4%)	24 (1.4%)	198 (1.5%)
Balance While Standing				
Maintained position as required in test	256 (4.9%)	403 (6.9%)	134 (7.8%)	793 (6.2%)
Unsteady, but able to rebalance self without physical support	709 (13.5%)	928 (15.9%)	290 (16.9%)	1927 (15.0%)
Partial physical support during test or stands but does not follow directions for test	1235 (23.5%)	1668 (28.5%)	517 (30.0%)	3420 (26.7%)
Not able to attempt test without physical help	3052 (58.1%)	2845 (48.7%)	780 (45.3%)	6677 (52.1%)
Balance While Sitting				
Maintained position as required in test	2846 (53.9%)	3857 (65.8%)	1188 (68.9%)	7891 (61.3%)
Unsteady, but able to rebalance self without physical support	665 (12.6%)	773 (13.2%)	212 (12.3%)	1650 (12.8%)
Partial physical support during test or stands but does not follow directions for test	873 (16.5%)	767 (13.1%)	205 (11.9%)	1845 (14.3%)
Not able to attempt test without physical help	897 (17.0%)	469 (8.0%)	120 (7.0%)	1486 (11.5%)
Unsteady Gait				
Unsteady Gait	2459 (46.4%)	3192 (54.3%)	911 (52.8%)	6562 (50.9%)
Fell in past 30 days	1580 (29.8%)	2080 (35.4%)	559 (32.4%)	4219 (32.7%)
Fell in past 31–180 days	1320 (24.9%)	1548 (26.4%)	471 (27.3%)	3339 (25.9%)
Hip fracture in last 180 days	304 (5.7%)	421 (7.2%)	92 (5.3%)	817 (6.3%)
Other fracture in last 180 days	346 (6.5%)	533 (9.1%)	154 (8.9%)	1033 (8.0%)
Number of meds in the last 7 days				
0 medications	9 (0.2%)	7 (0.1%)	1 (0.1%)	17 (0.1%)
1 to 5 medications	375 (7.1%)	376 (6.4%)	104 (6.0%)	855 (6.6%)
6 to 10 medications	1740 (32.8%)	1977 (33.7%)	580 (33.6%)	4297 (33.3%)
>10 medications	3.173 (59.9%)	3515 (59.8%)	1041 (60.3%)	7729 (59.9%)
Concomitant Medications				
Calcium Channel Blockers	1319 (24.9%)	16,26 (27.7%)	507 (29.4%)	3452 (26.8%)
Cognitive Enhancers	1449 (27.4%)	1576 (26.8%)	509 (29.5%)	3534 (27.4%)
Antiparkinson	536 (10.1%)	585 (10.0%)	208 (12.1%)	1329 (10.3%)
Diuretics	2378 (44.9%)	2722 (46.3%)	811 (47.0%)	5911 (45.8%)
Antipsychotics	1510 (28.5%)	1277 (21.7%)	363 (21.0%)	3150 (24.4%)
Antivertigo	1164 (22.0%)	769 (13.1%)	210 (12.2%)	2143 (16.6%)
Beta Blockers	2296 (43.3%)	2497 (42.5%)	778 (45.1%)	5571 (43.2%)
Anxiolytic Sedative/Hypnotic Agent	795 (15.0%)	892 (15.2%)	250 (14.5%)	1937 (15.0%)
ACE Inhibitors	1521 (28.7%)	1855 (31.6%)	538 (31.2%)	3914 (30.3%)
Antidepressant	3019 (57.0%)	3319 (56.5%)	1004 (58.2%)	7342 (56.9%)
ARB	506 (9.6%)	653 (11.1%)	244 (14.1%)	1403 (10.9%)
Alpha Blockers	758 (14.3%)	822 (14.0%)	286 (16.6%)	1866 (14.5%)
Anticonvulsant	1336 (25.2%)	1263 (21.5%)	411 (23.8%)	3010 (23.3%)
Vasodilators	815 (15.4%)	734 (12.5%)	234 (13.6%)	1783 (13.8%)
Benzodiazepines	45 (0.8%)	49 (0.8%)	15 (0.9%)	109 (0.8%)
Mean Anticholinergic Burden/ADS Score (SD)	2.8 (2.45)	4.2 (2.46)	2.1 (2.00)	3.3 (2.53)

Table 3 shows multivariate model results for the entire cohort. Important factors predicting type of BAM initiated included demographic characteristics as well as medical conditions and treatments, and the use of non-pharmacological interventions for bladder control (i.e., any scheduled toileting plan, indwelling catheter, or use of pads/briefs). Some of these factors were also relevant in the sex-stratified models (Table 4), but some were only significant in the model based on the entire population. For example, age group of aged 85 and older was predictive of less non-selective IR BAM initiation in the study population even though it was not found to be predictive in the sex-stratified specifications. Black race and balance while sitting were found to be significant (black race was associated with more non-selective IR BAM initiation; balance while sitting recorded as “Not able to attempt test without physical help” was associated with less non-selective IR BAM initiation) in the combined model though these were not significant in the female-only specification. The absence of non-pharmacological continence management strategies (i.e., use of pads/briefs, indwelling catheter) were found to be significant predictors of more non-selective ER BAM initiation in the combined model specification. Anticholinergic burden (ADS score) was found to be a significant predictor of non-selective ER (relative to non-selective IR) and non-selective IR (relative to selective) BAM initiation in each model specification.

**Table 3:** Predictors of Type of Bladder Antimuscarinics Initiation in All New Users (*n* = 12,251)

	New non-selective ER user vs. new non-selective IR user	New selective user vs. New non-selective IR user
	*OR (95% CI)*	*OR (95% CI)*
Age Group		
65 to 74	Ref.	Ref.
75 to 84	1.044 (0.919–1.186)	0.982 (0.826–1.167)
85 and Older	0.857 (0.752–0.977)^a^	0.888 (0.743–1.060)
Female	1.266 (1.130–1.418)^a^	1.321 (1.132–1.541)^a^
Race		
White	Ref.	Ref.
Black	1.288 (1.089–1.523)^a^	0.894 (0.703–1.137)
Other Race	1.062 (0.818–1.380)	1.353 (0.952–1.923)
Nursing Home Region		
Midwest	Ref.	Ref.
Northeast	0.624 (0.557–0.700)^a^	0.562 (0.476–0.664)^a^
South	0.647 (0.581–0.719)^a^	1.052 (0.917–1.207)
West	0.848 (0.708–1.016)	0.622 (0.474–0.815)^a^
Body Mass Index		
Underweight	0.779 (0.663–0.916)^a^	0.673 (0.527–0.859)^a^
Normal	Ref.	Ref.
Overweight	1.080 (0.971–1.202)	1.181 (1.024–1.362)^a^
Obese	1.346 (1.196–1.515)^a^	1.319 (1.124–1.547)^a^
Cognitive Performance Score		
Intact	Ref.	Ref.
Borderline intact/Mild Impairment	1.009 (0.895–1.137)	1.057 (0.900–1.240)
Moderate impairment	1.011 (0.886–1.154)	0.937 (0.783–1.120)
Moderate-severe/Severe/Very Severe impairment	0.549 (0.456–0.659)^a^	0.512 (0.391–0.669)^a^
CHESS		
Score of 0	Ref.	Ref.
Score of 1 or 2	0.940 (0.849–1.040)	0.853 (0.745–0.977)^a^
Score of 3	0.727 (0.612–0.864)^a^	0.837 (0.663–1.057)
Score of 4 or 5	0.685 (0.498–0.943)^a^	0.835 (0.538–1.295)
Activities of Daily Living	0.989 (0.980–0.997)^a^	0.976 (0.965–0.988)^a^
Elixhauser/Other Comorbidities		
Aphasia	0.554 (0.411–0.746)^a^	0.616 (0.399–0.951)^a^
Congestive Heart Failure	0.831 (0.745–0.927)^a^	0.834 (0.716–0.971)^a^
Hypertension, Uncomplicated	1.282 (1.170–1.406)^a^	1.037 (0.915–1.176)
Weight Loss	0.777 (0.652–0.926)^a^	0.772 (0.596–0.999)^a^
Bladder Continence		
Continent	Ref.	Ref.
Usually Continent	1.235 (1.064–1.433)^a^	1.076 (0.880–1.316)
Occasionally Incontinent	1.575 (1.355–1.830)^a^	1.495 (1.224–1.827)^a^
Frequently Incontinent	1.524 (1.326–1.751)^a^	1.503 (1.246–1.813)^a^
Incontinent	1.083 (0.909–1.290)	1.204 (0.936–1.550)
No Bladder Continence Appliance or Program	1.217 (1.081–1.371)^a^	0.982 (0.836–1.153)
Bowel Continence		
Continent	Ref.	Ref.
Usually Continent	0.741 (0.632–0.869)^a^	0.936 (0.763–1.150)
Occasionally Incontinent	0.938 (0.775–1.135)	0.802 (0.617–1.042)
Frequently Incontinent	0.759 (0.641–0.899)^a^	0.626 (0.493–0.795)^a^
Incontinent	0.688 (0.573–0.826)^a^	0.582 (0.446–0.758)^a^
Urinary Tract Infection in Last 30 Days	1.119 (1.006–1.244)^a^	1.197 (1.037–1.382)^a^
Gait		
Unsteady Gait	1.140 (1.042–1.246)^a^	1.017 (0.900–1.148)
Fell in Past 30 days	1.193 (1.082–1.315)^a^	1.072 (0.939–1.223)
Hip Fracture in Last 180 Days	1.254 (1.047–1.502)^a^	1.025 (0.778–1.333)
Other Fracture in Last 180 Days	1.249 (1.060–1.472)^a^	1.344 (1.071–1.662)^a^
Balance While Sitting		
Maintained position as required in test	Ref.	Ref.
Unsteady, but able to rebalance self without physical support	1.043 (0.911–1.193)	0.958 (0.796–1.152)
Partial physical support during test or stands but does not follow directions for test	1.026 (0.896–1.174)	0.866 (0.715–1.049)
Not able to attempt test without physical help	0.883 (0.709–0.980)^a^	0.700 (0.551–0.890)^a^
Concomitant Medications		
Calcium Channel Blockers	1.014 (0.919–1.120)	1.182 (1.036–1.349)^a^
Cognitive Enhancers	1.031 (0.929–1.144)	1.203 (1.047–1.382)^a^
Antiparkinson	0.896 (0.774–1.038)	1.372 (1.139–1.653)^a^
Antipsychotics	0.476 (0.425–0.532)^a^	0.874 (0.751–1.017)
Antivertigo	0.269 (0.236–0.307)^a^	0.727 (0.608–0.869)^a^
Anxiolytic Sedative or Hypnotic Agent	0.672 (0.591–0.763)^a^	1.015 (0.853–1.208)
Antidepressant	0.597 (0.543–0.656)^a^	1.315 (1.157–1.494)^a^
ARB	1.024 (0.889–1.180)	1.287 (1.077–1.538)^a^
Alpha Blockers	1.024 (0.894–1.173)	1.299 (1.088–1.553)^a^
Anticonvulsant	0.719 (0.646–0.799)^a^	0.935 (0.812–1.076)
Vasodilators	0.595 (0.523–0.676)^a^	1.024 (0.861–1.216)
Benzodiazepines	2.249 (1.357–3.727)^a^	1.694 (0.860–3.338)
Anticholinergic Burden Score	1.476 (1.443–1.510)^a^	0.808 (0.780–0.836)^a^

^a^Indicates statistical significance

**Table 4:** Predictors of Type of Bladder Antimuscarinics Initiation, Stratified by Sex

	Females (*n* = 9322)		Males (*n* = 3011)	
	New non-selective ER user vs. new non-selective IR user	New selective user vs. New non-selective IR user	New non-selective ER user vs. new non-selective IR user	New selective user vs. New non-selective IR user
	*OR (95% CI)*	*OR (95% CI)*	*OR (95% CI)*	*OR (95% CI)*
Race				
White	–	–	Ref.	Ref.
Black	–	–	1.771 (1.319–2.379)^a^	1.085 (0.708–1.664)
Other Race	–	–	1.092 (0.685–1.742)	1.930 (1.086–3.430)^a^
Nursing Home Region				
Midwest	Ref.	Ref.	Ref.	Ref.
Northeast	0.687 (0.603–0.782)^a^	0.607 (0.503–0.734)^a^	0.419 (0.329–0.534)^a^	0.422 (0.295–0.605)^a^
South	0.709 (0.629–0.799)^a^	1.074 (0.917–1.257)	0.528 (0.420–0.664)^a^	1.094 (0.823–1.453)
West	0.849 (0.689–1.047)	0.686 (0.501–0.940)^a^	0.886 (0.628–1.250)	0.550 (0.328–0.923)^a^
Body Mass Index				
Underweight	0.769 (0.643–0.919)^a^	0.700 (0.537–0.913)^a^	0.846 (0.583–1.227)	0.498 (0.262–0.949)^a^
Normal	Ref.	Ref.	Ref.	Ref.
Overweight	1.122 (0.992–1.269)	1.151 (0.974–1.359)	0.982 (0.791–1.218)	1.413 (1.065–1.876)^a^
Obese	1.402 (1.229–1.598)^a^	1.284 (1.073–1.537)^a^	1.398 (1.085–1.801)^a^	1.739 (1.251–2.418)^a^
Cognitive Performance Score				
Intact	Ref.	Ref.	Ref.	Ref.
Borderline intact/Mild Impairment	1.005 (0.878–1.150)	1.024 (0.854–1.228)	0.968 (0.747–1.255)	1.103 (0.782–1.558)
Moderate impairment	0.991 (0.853–1.150)	0.884 (0.722–1.084)	1.018 (0.772–1.342)	1.073 (0.740–1.556)
Moderate-severe/Severe/Very Severe impairment	0.523 (0.424–0.645)^a^	0.424 (0.310–0.579)^a^	0.519 (0.361–0.747)^a^	0.715 (0.432–1.185)
CHESS				
Score of 0	Ref.	Ref.	Ref.	Ref.
Score of 1 or 2	0.959 (0.855–1.077)	0.916 (0.783–1.071)	0.882 (0.716–1.086)	0.709 (0.539–0.932)^a^
Score of 3	0.793 (0.653–0.963)^a^	0.906 (0.693–1.185)	0.517 (0.354–0.754)^a^	0.683 (0.422–1.106)
Score of 4 or 5	0.666(0.468–0.948)^a^	0.673 (0.396–1.144)	0.657 (0.318–1.356)	1.639 (0.727–3.695)
Activities of Daily Living	0.989 (0.979–0.998)^a^	0.975 (0.963–0.988)^a^	0.983 (0.964–1.002)	0.969 (0.945–0.994)^a^
Elixhauser/Other Comorbidities				
Aphasia	0.564 (0.404–0.787)^a^	0.523 (0.313–0.903)^a^	–	–
Congestive Heart Failure	0.816 (0.720–0.925)^a^	0.804 (0.674–0.959)^a^	–	–
Hypertension, Uncomplicated	1.348 (1.213–1.497)^a^	1.105 (0.957–1.276)	–	–
Weight Loss	0.765 (0.622–0.942)^a^	0.767 (0.565–1.042)	–	–
Blood Loss Anemia	0.982 (0.709–1.361)	0.585 (0.348–0.982)^a^	–	–
Hemiplegia/Hemiparesis	–	–	1.581 (1.152–2.168)^a^	1.618 (1.075–2.435)^a^
Diabetes, Complicated	–	–	0.889 (0.615–1.284)	0.405 (0.215–0.766)^a^
Bladder Continence				
Continent	Ref.	Ref.	Ref.	Ref.
Usually Continent	1.144 (0.963–1.359)	0.999 (0.789–1.265)	1.543 (1.108–2.149)^a^	0.994 (0.640–1.543)
Occasionally Incontinent	1.570 (1.311–1.881)^a^	1.359 (1.063–1.737)^a^	1.601 (1.153–2.224)^a^	1.510 (0.994–2.294)
Frequently Incontinent	1.406 (1.182–1.672)^a^	1.389 (1.097–1.760)^a^	2.011 (1.484–2.726)^a^	1.264 (0.846–1.888)
Incontinent	1.048 (0.835–1.315)	1.100 (0.791–1.531)	1.282 (0.902–1.822)	1.055 (0.652–1.708)
Indwelling Catheter	1.028 (0.864–1.222)	0.774 (0.600–0.998)^a^	–	–
No Bladder Continence Appliance or Program	1.158 (1.002–1.339)^a^	0.917 (0.753–1.117)	–	–
Pads/Briefs Used	–	–	0.769 (0.602–0.982)^a^	1.271 (0.917–1.763)
Bowel Continence				
Continent	Ref.	Ref.	Ref.	Ref.
Usually Continent	0.750 (0.626–0.899)^a^	0.940 (0.742–1.191)	0.754 (0.535–1.061)	0.954 (0.622–1.463)
Occasionally Incontinent	0.981 (0.786–1.224)	0.812 (0.597–1.104)	0.855 (0.581–1.258)	0.783 (0.469–1.309)
Frequently Incontinent	0.771 (0.633–0.938)^a^	0.640 (0.483–0.848)^a^	0.737 (0.525–1.036)	0.596 (0.371–0.958)^a^
Incontinent	0.656 (0.529–0.813)^a^	0.564 (0.409–0.778)^a^	0.742 (0.519–1.062)	0.560 (0.340–0.920)^a^
Bowel Elimination Pattern: Regular	0.962 (0.854–1.084)	0.846 (0.720–0.995)^a^	–	–
Urinary Tract Infection in Last 30 Days	1.123 (0.997–1.265)	1.254 (1.067–1.474)^a^	–	–
Gait				
Unsteady Gait	1.156 (1.044–1.280)^a^	1.074 (0.935–1.234)	–	–
Fell in Past 30 days	1.153 (1.032–1.288)^a^	1.053 (0.904–1.226)	1.315 (1.077–1.607)^a^	1.063 (0.812–1.390)
Hip Fracture in Last 180 Days	1.297 (1.063–1.583)^a^	1.084 (0.811–1.449)	–	–
Other Fracture in Last 180 Days	1.230 (1.033–1.465)^a^	1.339 (1.059–1.694)^a^	–	–
Balance While Standing				
Maintained position as required in test	–	–	Ref.	Ref.
Unsteady, but able to rebalance self without physical support	–	–	0.572 (0.370–0.885)^a^	0.806 (0.465–1.396)
Partial physical support during test or stands but does not follow directions for test	–	–	0.766 (0.503–1.167)	0.879 (0.513–1.506)
Not able to attempt test without physical help	–	–	0.777 (0.500–1.208)	0.896 (0.506–1.588)
Balance While Sitting				
Maintained position as required in test	–	–	Ref.	Ref.
Unsteady, but able to rebalance self without physical support	–	–	0.968 (0.719–1.302)	1.259 (0.871–1.819)
Partial physical support during test or stands but does not follow directions for test	–	–	0.883 (0.662–1.178)	0.702 (0.463–1.064)
Not able to attempt test without physical help	–	–	0.650 (0.466–0.909)^a^	0.580 (0.356–0.945)^a^
Concomitant Medications				
Calcium Channel Blockers	1.025 (0.917–1.145)	1.195 (1.031–1.386)^a^	–	–
Cognitive Enhancers	1.057 (0.939–1.191)	1.280 (1.091–1.501)^a^	–	–
Antiparkinson	0.891 (0.749–1.059)	1.334 (1.066–1.668)^a^	0.957 (0.727–1.259)	1.462 (1.039–2.056)^a^
Antipsychotics	0.500 (0.440–0.567)^a^	0.875 (0.735–1.043)	0.440 (0.349–0.556)^a^	0.891 (0.657–1.207)
Antivertigo	0.296 (0.256–0.342)^a^	0.763 (0.624–0.932)^a^	0.174 (0.127–0.237)^a^	0.642 (0.430–0.960)^a^
Anxiolytic Sedative or Hypnotic Agent	0.671 (0.580–0.775)^a^	1.070 (0.879–1.302)	0.723 (0.548–0.952)^a^	0.911 (0.625–1.327)
Antidepressant	0.585 (0.526–0.651)^a^	1.326 (1.145–1.535)^a^	0.617 (0.508–0.952)^a^	1.313 (1.012–1.704)^a^
ARB	1.066 (0.913–1.244)	1.340 (1.103–1.629)^a^	–	–
Alpha Blockers	0.763 (0.622–0.935)^a^	0.893 (0.678–1.176)	1.286 (1.067–1.549)^a^	1.752 (1.372–2.237)^a^
Anticonvulsant	0.733 (0.650–0.826)^a^	0.941 (0.801–1.106)	0.755 (0.607–0.940)^a^	0.965 (0.726–1.283)
Vasodilators	0.609 (0.526–0.705)^a^	1.071 (0.879–1.306)	0.508 (0.390–0.663)^a^	0.912 (0.641–1.297)
Benzodiazepines	1.968 (1.099–3.526)^a^	2.232 (1.085–4.592)^a^	3.592 (1.331–9.690)^a^	Omission (Not enough data)
Anticholinergic Burden Score	1.455 (1.418–1.493)^a^	0.798 (0.767–0.830)^a^	1.578 (1.501–1.659)^a^	0.846 (0.786–0.911)^a^

^a^Indicates statistical significance

Table 4 shows the results of the multivariate models, stratified by sex. Region of nursing home, BMI, cognitive performance score, CHESS, ADLs, and bladder continence were significant predictors of BAM in both sexes. Age and number of medications were not a significant factor in type of new BAM use in either sex. A history of fractures and fall-related injuries were significant predictors of type of BAM use in females (specifically, unsteady gait, hip fracture in last 180 days, and other fracture in last 180 days, all of which were associated with more non-selective ER relative to non-selective IR), but not in males. However, indicators of balance issues were significant predictors of less non-selective ER relative to non-selective IR BAM use in males. Fewer co-morbidities and concomitant medications were significant predictors of type of BAM use in males when compared to females. Significant predicting comorbidities in females included aphasia (less non-selective IR), congestive heart failure (less non-selective IR), hypertension (uncomplicated; more non-selective IR), weight loss (more non-selective IR), and blood loss anemia (less non-selective IR), and none of these were predictors in males. Hemiplegia/hemiparesis (more non-selective IR) as well as diabetes (complicated; less non-selective IR) were significant in males, but not in females. All significant predicting concomitant medications were found in both females and in males with the exception of calcium channel blockers, cognitive enhancers, and ARB, which were not found to be significant predictors in males. Interestingly, race played a predictive role in type of BAM initiation in males (less non-selective IR), but not in females.

### Sensitivity analyses

A specification using imputed values for the full model with no stratification by sex was analyzed to further test sensitivity (see Table 5). When imputed values were used for the full model specification, estimates for race, balance while sitting, and aphasia were found to be somewhat different from estimates calculated without imputation. In the full specification, fracture injuries (hip and other) were associated with smaller estimates as predictors of type of BAM initiation. Unlike the specifications where sex was stratified, the full model specification did not result in larger estimate sizes for non-pharmacological continence management variables. Overall, the similarity of results between the original model and sensitivity analyses using imputed values suggests that the original model is robust.

**Table 5:** Predictors of Type of Bladder Antimuscarinics Initiation Using Imputed Values in All New Users (*n* = 12,899)

	New non-selective ER user vs. New non-selective IR user	New selective user vs. new non-selective IR user
	*OR (95% CI)*	*OR (95% CI)*
Age Group		
65 to 74	Ref.	Ref.
75 to 84	1.019 (0.900–1.153)	0.956 (0.808–1.130)
85 and Older	0.863 (0.760–0.980)^a^	0.884 (0.745–1.050)
Female	1.279 (1.145–1.428)^a^	1.324 (1.139–1.539)^a^
Race		
White	Ref.	Ref.
Black	1.269 (1.078–1.494)^a^	0.925 (0.734–1.165)
Other Race	1.019 (0.789–1.316)	1.269 (0.898–1.793)
Nursing Home Region		
Midwest	Ref.	Ref.
Northeast	0.627 (0.561–0.700)^a^	0.575 (0.489–0.675)^a^
South	0.651 (0.587–0.723)	1.043 (0.911–1.193)
West	0.868 (0.727–1.036)	0.630 (0.483–0.821)^a^
Body Mass Index		
Underweight	0.785 (0.670–0.919)^a^	0.684 (0.539–0.869)^a^
Normal	Ref.	Ref.
Overweight	1.085 (0.978–1.204)	1.198 (1.042–1.378)^a^
Obese	1.322 (1.178–1.483)^a^	1.342 (1.149–1.567)^a^
Cognitive Performance Score		
Intact	Ref.	Ref.
Borderline intact/Mild Impairment	1.006 (0.896–1.131)	1.071 (0.916–1.252)
Moderate impairment	0.997 (0.876–1.134)	0.943 (0.792–1.123)
Moderate-severe/Severe/Very Severe impairment	0.526 (0.440–0.628)^a^	0.527 (0.406–0.683)^a^
CHESS		
Score of 0	Ref.	Ref.
Score of 1 or 2	0.928 (0.840–1.025)	0.852 (0.746–0.974)^a^
Score of 3	0.729 (0.615–0.865)^a^	0.820 (0.650–1.034)
Score of 4 or 5	0.650 (0.475–0.890)^a^	0.811 (0.527–1.248
Activities of Daily Living	0.989 (0.980–0.997)^a^	0.976 (0.965–0.987)^a^
Elixhauser/Other Comorbidities		
Aphasia	0.605 (0.457–0.801)^a^	0.574 (0.373–0.882)^a^
Congestive Heart Failure	0.839 (0.755–0.934)^a^	0.865 (0.747–1.002)
Hypertension, Uncomplicated	1.295 (1.184–1.416)^a^	1.054 (0.933–1.191)
Weight Loss	0.753 (0.635–0.893)^a^	0.745 (0.580–0.958)^a^
Bladder Continence		
Continent	Ref.	Ref.
Usually Continent	1.244 (1.076–1.438)^a^	1.099 (0.903–1.337)
Occasionally Incontinent	1.586 (1.370–1.837)^a^	1.494 (1.228–1.819)^a^
Frequently Incontinent	1.538 (1.343–1.761)^a^	1.517 (1.263–1.823)^a^
Incontinent	1.083 (0.915–1.281)	1.240 (0.973–1.582)
No Bladder Continence Appliance or Program	1.214 (1.081–1.363)^a^	0.997 (0.852–1.166)
Bowel Continence		
Continent	Ref.	Ref.
Usually Continent	0.729 (0.624–0.852)^a^	0.943 (0.771–1.153)
Occasionally Incontinent	0.901 (0.748–1.086)	0.768 (0.593–0.994)^a^
Frequently Incontinent	0.750 (0.636–0.885)^a^	0.641 (0.508–0.809)^a^
Incontinent	0.678 (0.568–0.808)^a^	0.583 (0.451–0.753)^a^
Urinary Tract Infection in Last 30 Days	1.104 (0.995–1.225)	1.168 (1.015–1.344)^a^
Gait		
Unsteady Gait	1.019 (0.894–1.162)	0.939 (0.784–1.124)
Fell in Past 30 days	1.032 (0.904–1.177)	0.890 (0.739–1.071)
Hip Fracture in Last 180 Days	0.825 (0.705–0.966)^a^	0.679 (0.538–0.857)^a^
Other Fracture in Last 180 Days	1.131 (1.037–1.235)^a^	1.025 (0.911–1.155)
Balance While Sitting		
Maintained position as required in test	Ref.	Ref.
Unsteady, but able to rebalance self without physical support	1.183 (1.076–1.300)^a^	1.064 (0.935–1.210)
Partial physical support during test or stands but does not follow directions for test	0.252 (1.049–1.494)^a^	1.057 (0.818–1.365)
Not able to attempt test without physical help	1.250 (1.065–1.467)^a^	1.348 (1.090–1.668)^a^
Concomitant Medications		
Calcium Channel Blockers	1.029 (0.935–1.134)	1.156 (1.016–1.315)^a^
Cognitive Enhancers	1.049 (1.044–1.369)^a^	1.196 (1.044–1.369)^a^
Antiparkinson	0.902 (0.782–1.040)	1.354 (1.129–1.625)^a^
Antipsychotics	0.472 (0.423–0.526)^a^	0.873 (0.753–1.012)
Antivertigo	0.267 (0.235–0.303)^a^	0.729 (0.612–0.867)^a^
Anxiolytic Sedative or Hypnotic Agent	0.671 (0.592–0.760)^a^	1.025 (0.865–1.214)
Antidepressant	0.602 (0.549–0.660)^a^	1.301 (1.148–1.474)^a^
ARB	1.004 (0.874–1.152)	1.305 (1.097–1.552)^a^
Alpha Blockers	1.038 (0.909–1.186)	1.327 (1.116–1.579)^a^
Anticonvulsant	0.727 (0.656–0.806)^a^	0.930 (0.810–1.067)
Vasodilators	0.584 (0.515–0.662)^a^	0.990 (0.836–1.172)
Benzodiazepines	2.058 (1.251–3.387)^a^	1.885 (0.993–3.578)
Anticholinergic Burden Score	1.478 (1.445–1.511)^a^	0.807 (0.780–0.835)^a^

^a^Indicates statistical significance

Sensitivity analysis using imputed values revealed similar results for both the female and male model specifications (see Table 6). However, when imputed values were used estimates for type of BAM initiation were found to be smaller for each of the following predictor variables: race, aphasia (in females), hemiplegia/hemiparesis (in males), diabetes (in males), urinary tract infection, balance while sitting, fell in past 30 days (in females), anti-Parkinson’s medications, anti-vertigo medications, antidepressants, and benzodiazepines. Conversely, non-pharmacological approaches to incontinence management were found to yield larger estimates on type of BAM initiation when imputed values were used. Specifically, estimates were larger for use of pads or briefs as well as regular bowel elimination patterns.

**Table 6:** Predictors of Type of Bladder Antimuscarinics Initiation Using Imputed Values, Stratified by Sex

	Females (*n* = 9732)		Males (*n* = 3167)	
	New non-selective ER user vs. new non-selective IR user	New selective user vs. New non-selective IR user	New non-selective ER user vs. new non-selective IR user	New selective user vs. New non-selective IR user
	*OR (95% CI)*	*OR (95% CI)*	*OR (95% CI)*	*OR (95% CI)*
Race				
White	–	–	Ref.	Ref.
Black	–	–	1.732 (1.301–2.305)^a^	1.020 (0.673–1.547)
Other Race	–	–	1.092 (0.692–1.723)	1.769 (1.004–3.118)^a^
Nursing Home Region				
Midwest	Ref.	Ref.	Ref.	Ref.
Northeast	0.688 (0.606–0.781)^a^	0.604 (0.503–0.727)^a^	0.426 (0.337–0.539)^a^	0.478 (0.340–0.672)^a^
South	0.709 (0.630–0.797)^a^	1.047 (0.897–1.222)	0.534 (0.428–0.667)^a^	1.101 (0.834–1.454)
West	0.857 (0.698–1.053)	0.660 (0.483–0.903)^a^	0.912 (0.651–1.278)	0.603 (0.366–0.993)^a^
Body Mass Index				
Underweight	0.781 (0.656–0.931)^a^	0.727 (0.561–0.943)^a^	0.816 (0.570–1.170)	0.442 (0.234–0.837)^a^
Normal	Ref.	Ref.	Ref.	Ref.
Overweight	1.130 (1.002–1.276)^a^	1.184 (1.006–1.394)^a^	0.982 (0.795–1.211)	1.365 (1.038–1.796)^a^
Obese	1.364 (1.200–1.551)^a^	1.347 (1.131–1.605)^a^	1.394 (1.090–1.783)^a^	1.654 (1.201–2.278)^a^
Cognitive Performance Score				
Intact	Ref.	Ref.	Ref.	Ref.
Borderline intact/Mild Impairment	1.008 (0.883–1.150)	1.061 (0.889–1.267)	0.960 (0.747–1.235)	1.016 (0.729–1.417)
Moderate impairment	0.978 (0.845–1.132)	0.891 (0.730–1.088)	1.009 (0.772–1.320)	1.018 (0.711–1.457)
Moderate-severe/Severe/Very Severe impairment	0.508 (0.414–0.624)^a^	0.445 (0.328–0.603)^a^	0.509 (0.358–0.723)^a^	0.689 (0.424–1.119)
CHESS				
Score of 0	Ref.	Ref.	Ref.	Ref.
Score of 1 or 2	0.951 (0.849–1.066)	0.906 (0.776–1.057)	0.871 (0.710–1.069)	0.727 (0.557–0.949)^a^
Score of 3	0.788 (0.650–0.956)^a^	0.882 (0.675–1.152)	0.525 (0.361–0.764)^a^	0.684 (0.423–1.103)
Score of 4 or 5	0.648 (0.457–0.920)^a^	0.691 (0.412–1.159)	0.612 (0.299–1.252)	1.431 (0.639–3.204)
Activities of Daily Living	0.990 (0.980–0.999)^a^	0.975 (0.963–0.988)^a^	0.984 (0.965–1.002)	0.972 (0.948–0.997)^a^
Elixhauser/Other Comorbidities				
Aphasia	0.574 (0.416–0.790)^a^	0.496 (0.293–0.840)^a^	–	–
Congestive Heart Failure	0.814 (0.720–0.920)^a^	0.811 (0.683–0.963)^a^	–	–
Hypertension, Uncomplicated	1.348 (1.216–1.494)^a^	1.108 (0.962–1.275)	–	–
Weight Loss	0.752 (0.614–0.920)^a^	0.746 (0.552–1.007)	–	–
Blood Loss Anemia	0.987 (0.718–1.357)	0.596 (0.359–0.988)^a^	–	–
Hemiplegia/Hemiparesis	–	–	1.547 (1.139–2.100)^a^	1.598 (1.075–2.376)^a^
Diabetes, Complicated	–	–	0.940 (0.657–1.344)	0.454 (0.250–0.826)^a^
Bladder Continence				
Continent	Ref.	Ref.	Ref.	Ref.
Usually Continent	1.157 (0.978–1.370)	1.028 (0.816–1.295)	1.476 (1.070–2.035)^a^	0.988 (0.645–1.512)
Occasionally Incontinent	1.536 (1.288–1.833)^a^	1.346 (1.058–1.712)^a^	1.663 (1.295–2.295)^a^	1.442 (0.957–2.174)
Frequently Incontinent	1.400 (1.181–1.659)^a^	1.403 (1.113–1.770)^a^	2.032 (1.509–2.737)^a^	1.269 (0.859–1.875)
Incontinent	1.018 (0.815–1.270)	1.120 (0.812–1.543)	1.274 (0.908–1.786)	0.987 (0.620–1.571)
Indwelling Catheter	1.006 (0.849–1.191)	0.769 (0.601–0.985)^a^	–	–
Pads/Briefs Used	–	–	0.793 (0.625–1.006)	1.286 (0.935–1.767)
No Bladder Continence Appliance or Program	1.156 (1.003–1.333)^a^	0.935 (0.771–1.134)	–	–
Bowel Continence				
Continent	Ref.	Ref.	Ref.	Ref.
Usually Continent	0.741 (0.620–0.885)^a^	0.934 (0.740–1.178)	0.719 (0.515–1.004)	0.977 (0.647–1.475)
Occasionally Incontinent	0.935 (0.753–1.161)	0.779 (0.576–1.055)	0.832 (0.569–1.218)	0.764 (0.460–1.269)
Frequently Incontinent	0.766 (0.631–0.929)^a^	0.649 (0.493–0.854)^a^	0.728 (0.522–1.014)	0.595 (0.374–0.945)^a^
Incontinent	0.659 (0.535–0.813)^a^	0.575 (0.421–0.785)^a^	0.731 (0.517–1.033)	0.569 (0.352–0.921)^a^
Bowel Elimination Pattern: Regular	0.977 (0.869–1.098)	0.862 (0.736–1.010)	–	–
Urinary Tract Infection in Last 30 Days	1.118 (0.996–1.256)	1.217 (1.039–1.426)^a^	–	–
Balance While Standing				
Maintained position as required in test	–	–	Ref.	Ref.
Unsteady, but able to rebalance self without physical support	–	–	0.591 (0.387–0.904)^a^	0.738 (0.436–1.248)
Partial physical support during test or stands but does not follow directions for test	–	–	0.769 (0.511–1.157)	0.827 (0.495–1.381)
Not able to attempt test without physical help	–	–	0.786 (0.512–1.206)	0.838 (0.486–1.445)
Balance While Sitting				
Maintained position as required in test	–	–	Ref.	Ref.
Unsteady, but able to rebalance self without physical support	–	–	0.918 (0.688–1.224)	1.182 (0.827–1.690)
Partial physical support during test or stands but does not follow directions for test	–	–	0.878 (0.664–1.162)	0.679 (0.454–1.017)
Not able to attempt test without physical help	–	–	0.601 (0.434–0.831)^a^	0.532 (0.331–0.855)^a^
Gait				
Unsteady Gait	1.147 (1.038–1.267)^a^	1.087 (0.949–1.245)	–	–
Fell in Past 30 days	1.137 (1.020–1.266)^a^	1.050 (0.906–1.219)	1.349 (1.10–1.639)^a^	1.071 (0.825–1.391)
Hip Fracture in Last 180 Days	1.277 (1.050–1.554)^a^	1.117 (0.842–1.482)	–	–
Other Fracture in Last 180 Days	1.228 (1.035–1.457)^a^	1.331 (1.058–1.673)^a^	–	–
Concomitant Medications				
Calcium Channel Blockers	1.035 (0.929–1.154)	1.177 (1.018–1.361)^a^	–	–
Cognitive Enhancers	1.079 (0.960–1.212)	1.282 (1.097–1.499)^a^	–	–
Antiparkinson	0.894 (0.755–1.060)	1.327 (1.066–1.652)^a^	0.968 (0.741–1.266)	1.416 (1.014–1.978)^a^
Antipsychotics	0.490 (0.433–0.555)^a^	0.878 (0.722–1.019)	0.455 (0.363–0.571)^a^	0.928 (0.692–1.245)
Antivertigo	0.292 (0.254–0.337)^a^	0.791 (0.650–0.962)^a^	0.179 (0.132–0.242)^a^	0.573 (0.385–0.853)^a^
Anxiolytic Sedative or Hypnotic Agent	0.668 (0.580–0.770)^a^	1.074 (0.887–1.301)	0.710 (0.543–0.928)^a^	0.881 (0.609–1.275)
Antidepressant	0.593 (0.534–0.658)^a^	1.311 (1.136–1.513)^a^	0.598 (0.494–0.723)^a^	1.277 (0.991–1.646)
ARB	1.045 (0.898–1.216)	1.337 (1.104–1.619)^a^	–	–
Alpha Blockers	0.778 (0.637–0.950)^a^	0.921 (0.703–1.206)	1.288 (1.074–1.544)^a^	1.785 (1.408–2.264)^a^
Anticonvulsant	0.740 (0.658–0.833)^a^	0.931 (0.794–1.091)	0.750 (0.607–0.927)^a^	0.970 (0.737–1.277)
Vasodilators	0.600 (0.520–0.693)^a^	1.043 (0.859–1.268)	0.516 (0.398–0.669)^a^	0.875 (0.618–1.239)
Benzodiazepines	1.821 (1.022–3.245)^a^	2.327 (1.156–4.685)^a^	3.108 (1.168–8.269)^a^	0.573 (0.068–4.790)
Anticholinergic Burden Score	1.458 (1.422–1.495)^a^	0.796 (0.766–0.828)^a^	1.569 (1.495–1.647)^a^	0.846 (0.787–0.909)^a^

^a^Indicates statistical significance

Pearson tests were significant for both female and male models without imputation (*p* = 0.035 and *p* = 0.004, respectively) and with imputation (*p* = 0.034 and *p* = 0.002, respectively), which indicated good model fit. Likelihood ratio tests were also significant for both female and male models without imputation (*p* < 0.001 and *p* < 0.001, respectively) and with imputation (*p* < 0.001 and *p* < 0.001, respectively), which indicated joint significance of predictors for each model. The validation procedure for the model containing only females resulted in correct classification of 61.08% observations, meaning a majority match between predicted and observed values, and the validation procedure conducted on the model containing only males resulted in a correct classification of 64.34% observations.

## Discussion

To our knowledge, this was the first study to investigate factors associate with BAM initiation and to evaluate sex differences in BAM initiation based on receptor selectivity. In addition to informing the design of future comparative effectiveness studies of different BAM, our findings are clinically relevant for at least two reasons: first, the evidence that BAM type (i.e., selectivity) is associated with differential effects on cognitive function, and second, there are sex differences in prevalence and symptomatology of urinary incontinence. Specifically, these findings indicated that fall-related injuries were significant predictors of type of BAM initiation in females, while measures of balance were found to be significant predictors of type of BAM initiation in males. No non-pharmacological continence management strategy (i.e., use of pads/briefs, indwelling catheter) were found to be significant predictors of type of BAM initiation in the combined model specification, but in model specifications stratified by sex the use of pads/briefs was predictive of type of BAM initiation in males and use of indwelling catheter was predictive of type of BAM initiation in females. This suggests that there is little discernable influence of non-pharmacological continence management techniques on BAM prescribing practices, or at least that other factors are weighted more heavily in prescribing decisions.

Additionally, taking benzodiazepines concomitantly was a significant predictor of type of BAM initiation in both the combined model, and the sex-stratified model. Furthermore, in both the models, those with moderate to severe cognitive impairment were less likely to have been initiated on selective BAMs. This is of note, keeping in mind that selective BAMs may have a less harmful adverse event profile in comparison to non-selective BAMs. Severity of cognitive impairment does not seem to be associated with type of BAM initiation when pharmacological treatment options were considered.

The findings also indicate that prescribing preferences for type of BAM when BAM is initiated in nursing home patients may be, in part, driven by geography. Prescriptions for non-selective IR BAM were more likely than prescriptions for non-selective ER BAM and selective BAM in the Midwest region for both sexes, when compared to other regions. There is some evidence to suggest that prescribing patterns differ by geographic location for use of certain classes of medications (e.g., antipsychotic medications) in nursing home populations [53], though this has not been previously studied for BAM. One possible explanation for this geographic variation is range Pharmacy Benefit Management penetration across the regions, which could selectively influence type of BAM initiation by formulary structure. Previous research by Smith et al. has shown that Black nursing home residents are more likely to be at nursing homes that are greatly understaffed relative to the acuity profile of their residents [54]. This is important for conditions like urinary incontinence, where behavioral interventions need individual attention from the staff, and lacking this, pharmacological interventions may be relied upon. Svarstad et al. showed that treatment cultures at various nursing homes influence their efforts to reduce the use of psychotropic medications [55]. While these are not the class of drugs under discussion in our paper, it can be reasonably inferred that treatment cultures would influence the use of BAMs as well, especially considering the fact that they are adjunct therapy to be used in addition to other behavioral interventions. Although we did not observe any particular racial variation in the initiation by type of BAMs, differences in treatment cultures and racial disparities in nursing home care in the Midwest region may have driven the geographic variation.

Some limitations of the study included the use of claims data to identify users of these medications. Claims data are not recorded for research purposes, and therefore there could be miscoding errors. In addition, pharmacy claims lack the information on the diagnosis driving the indication; considering that some of the medications included in our study could be used for other purposes there is some room for misclassification. This is, however, to our knowledge, the first study that evaluates predictors of the initiation of BAM therapy by type, and provides insight on prescribing variability by various factors that could be used to further investigate appropriateness of medication utilization and conduct comparative effectiveness studies. There are also limitations regarding the extent of the analysis; namely, type of provider (e.g., MD or NP) prescribing BAM was not examined and transdermal BAM formulations were excluded form the analysis. Lastly, BAM formulations newer to the market (and therefore not summarized in Additional file 1: Table S1) may not have achieved widespread dissemination prior to the study period (e.g., extended release trospium was brought to market in 2007 at the beginning of the study period), and other changes in prescribing trends may have occurred in the time following the study period. Considering the descriptive nature of the current study, it would be difficult to make further inferences about the associations noted in our results. Certainly, this study raises important questions, including the predictive effect on BAM initiation with benzodiazepine use, or the noted geographic variability. However, further investigations would be necessary to completely understand and untangle these observations.

## Conclusions

Our investigation reveals important predictors of different BAM initiation in long-term care and describes differences between women and men. Our findings can be used to guide study design to develop and inform appropriate strategies to control for confounding in comparative effectiveness studies of different BAM; these are necessary steps for informing the clinical decision making process for BAM therapy selection in the nursing homes population and can be used to further develop strategies for targeted interventions to improve medication use for the treatment of urinary incontinence by addressing modifiable factors associated with BAM initiation.

## Additional file

Additional file 1: Table S1. Bladder Antimuscarinics- Important Drug Characteristics and Relevant Clinical Information. This supplementary table contains a summary of the important strengths and weaknesses of each bladder antimuscarinic medication included within this study, as well as a description of each medications’ M_1_-M_5_ receptor affinity. (DOCX 24 kb)

## Abbreviations

ACE: Angiotensin-converting-enzyme
ADL: Activities of Daily Living
ARB: Angiotensin-receptor-blocker
BAM: Bladder antimuscarinics
BMI: Body mass index
CHESS: Changes in Health, End-Stage Disease and Symptoms and Signs
ER: Extended release
GEE: Generalized estimating equations
HMO: Health Maintenance Organization
IR: Immediate release
MDS: Minimum Data Sets
QOL: Quality of life
