# Supplementary material for: An investigation of factors predicting the type of bladder antimuscarinics initiated in Medicare nursing homes residents
Source: BMC Geriatr. 2017 Dec 28;17:295. doi: 10.1186/s12877-017-0690-2 (PMC5745609; doi:10.1186/s12877-017-0690-2)
Supplement: Additional file 1: Table S1. — Bladder Antimuscarinics- Important Drug Characteristics and Relevant Clinical Information. This supplementary table contains a summary of the important strengths and weaknesses of each bladder antimuscarinic medication included within this study, as well as a description of each medications’ M1-M5 receptor affinity. (DOCX 24 kb) [file 12877_2017_690_MOESM1_ESM.docx]

**Additional file 1: Table S1.** Bladder Antimuscarinics- Important Drug Characteristics and Relevant Clinical Information [34, 37-45]

| **Bladder antimuscarinic** | **M_1_-M_5_ receptor affinity*** | **Other important characteristics**** | **Relative strengths and weaknesses** |
| --- | --- | --- | --- |
| Oxybutynin | Preferential binding to M_1_ and M_3_ receptors  No selectivity for M_3_ compared to M_1_  Selectivity for M_3_ compared to M_2_, M_4_, or M_5_ | Highly lipophilic tertiary amine with low molecular weight (<400 kDa)  Mixed action: muscarinic M_1_/M_3_ receptor antagonist, calcium antagonist and local anesthetic actions  Potent inhibitor of CYP3A4 | Penetrates the CNS, thus increasing the risk of cognitive effects; CNS effects reported in the literature  Dry mouth, constipation, headache and blurred vision reported frequently in RCTs |
| Tolterodine | Does not discriminate between different muscarinic receptors  In animal models, greater bladder-to-salivary gland selectivity ratios have been reported for tolterodine compared to oxybutynin | Tertiary amine  Molecular weight <400 kDa (tartrate >400)  Low-moderate lipophilic effects  Metabolized via CYP450 system | Dry mouth, constipation, headache and blurred vision reported frequently in RCTs  Minimal effects on quantitative EEG measures  Case reports of night terrors, effects on memory and hallucinations  Concomitant administration with CYP3A4 or 2D6 inhibitors or other drugs metabolized via P450 enzymes should be avoided |
| Trospium | Does not discriminate between different M receptors | Quaternary ammonium compound  Molecular weight <400 kDa (chloride >400)  Very low (hydrophilic) lipophilic effect, high polarity  Renal excretion | Limited access to the CNS  Dry mouth, constipation, headache and blurred vision reported frequently in RCTs  Minimal effects on quantitative EEG measures  Not metabolized by the CYP450 system in the liver, which minimizes the likelihood of any potentially harmful drug interactions with this agent. |
| Propantheline | Non selective | Quaternary ammonium compound; poorly absorbed after oral administration (<15%) and food significantly reduces bioavailability  Mixed action: antimuscarinic and ganglionic-blocking effects | Confusion, agitation and orthostatic hypotension reported frequently  High incidence of side effects, especially in the elderly. |
| Flavoxate | Non selective affinity | Pharmacological effect mediated by an active metabolite, MFCA with good oral bioavailability  Mixed action: antimuscarinic effect as well as direct spasmolytic effect | Clinical efficacy data is weak |
| Hyoscyamine | Non selective affinity |  | Clinical efficacy data is weak  High risk of systemic effects |
| Darifenacin | High selectivity for M_3_ compared to any other muscarinic receptor  In animal models, greater bladder-to-salivary gland selectivity ratios have been reported for darifenacin compared to oxybutynin | Tertiary amine  Molecular weight >400 kDa  Moderate lipophilic effect, positive polarity  Metabolized via CYP450 system | Dry mouth, constipation, headache and blurred vision reported frequently in RCTs  No CNS effects reported  Concomitant administration with CYP3A4 or 2D6 inhibitors or other drugs metabolized via these enzymes should be avoided |
| Solifenacin | M_3_ selectivity over M_2_  In animal models, greater bladder-to-salivary gland selectivity ratios have been reported for solifenacin compared to oxybutynin | Tertiary amine  Molecular weight <400 kDa ( succinate >400)  Low-moderate lipophilic effect  Potent inhibitor of CYP3A4 | Dry mouth, constipation, headache and blurred vision reported frequently in RCTs  No CNS effects reported |

*M_2_ and M_3_ receptors are the main muscarinic receptors involved in bladder control, while M_1_ receptors are involved in cognitive function; M_3_ receptors from salivary glands, lower bowel, and ciliary smooth muscle are related to BAM adverse effects: dry mouth, constipation, blurred vision. BAM agents with greater selectivity for the M_3_ receptors over the M_1_ receptors theoretically have less potential to cause cognitive impairment.

**Molecular size, lipophilicity and degree of ionization are important drug characteristics affecting penetration through the blood-brain barrier and the potential for cognitive effects. Molecules with molecular weight > 400 kDa cannot normally pass through the blood-brain barrier (Note: blood-brain barrier permeability is altered in older adults). For smaller molecules, the greater the lipid solubility, the more likely the molecule is to cross the blood-brain barrier; hydrophilic molecules do not pass the blood-brain barrier. In addition, the blood-brain barrier is less permeable to molecules with a neutral charge (low degree of ionization). In general, tertiary compounds have higher lipophilicity and molecular charge than quaternary agents; they are generally well absorbed from the gastrointestinal tract and are able to pass into the CNS, dependent on their individual physicochemical properties. Quaternary ammonium compounds are not well absorbed, pass into the CNS to a limited extent, and have a low incidence of CNS side-effects. They still produce peripheral antimuscarinic side-effects.
